# Polyuria in COVID-19 Patients Undergoing Extracorporeal Membrane Oxygenation

**DOI:** 10.3390/jcm13144081

**Published:** 2024-07-12

**Authors:** Johannes Rausch, Andrea U. Steinbicker, Benjamin Friedrichson, Armin N. Flinspach, Kai Zacharowski, Elisabeth H. Adam, Florian Piekarski

**Affiliations:** 1 Goethe University Frankfurt, University Hospital, Department of Anaesthesiology, Intensive Care Medicine and Pain Therapy, 60590 Frankfurt, Germany; 2 Department of Anesthesiology and Intensive Care Medicine, University Hospital Bonn, 53127 Bonn, Germany

**Keywords:** ECMO, polyuria, COVID-19, ARDS

## Abstract

**Background**: The COVID-19 pandemic caused an unprecedented number of patients requiring veno-venous extracorporeal membrane oxygenation (VV ECMO) therapy. Clinical polyuria was observed at our ECMO center during the pandemic. This study aims to investigate the incidence, potential causes, and implications of polyuria in COVID-19 patients undergoing VV ECMO therapy. **Methods**: Here, 68 SARS-CoV-2 positive patients receiving VV ECMO were stratified into the following two groups: polyuria (PU), characterized by an average urine output of ≥3000 mL/day within seven days following initiation, and non-polyuria (NPU), defined by <3000 mL/day. Polyuria in ECMO patients occurred in 51.5% (n = 35) within seven days after ECMO initiation. No significant difference in mortality was observed between PU and NPU groups (60.0% vs. 60.6%). Differences were found in the fluid intake (*p* < 0.01) and balance within 24 h (*p* = 0.01), creatinine (*p* < 0.01), plasma osmolality (*p* = < 0.01), lactate (*p* < 0.01), urea (*p* < 0.01), and sodium levels (*p* < 0.01) between the groups. Plasma osmolality increased (*p* < 0.01) after ECMO initiation during the observation period. **Results**: Diuresis and plasma osmolality increased during VV ECMO treatment, while mortality was not affected by polyuria. **Conclusions**: Polyuria does not appear to impact mortality. Further investigations are warranted to elucidate its underlying mechanisms and clinical implications in the context of VV ECMO therapy and COVID-19 management.

## 1. Introduction

Starting in early 2020, the world faced the coronavirus disease 2019 (COVID-19) pandemic. One severe complication of COVID-19 is acute respiratory distress syndrome (ARDS). Veno-venous extracorporeal membrane oxygenation (VV ECMO) therapy is a well-established treatment for ARDS [1]. VV ECMO offers artificial oxygenation in patients with respiratory failure. The COVID-19 pandemic has resulted in an unprecedented number of patients requiring VV ECMO. The Extracorporeal Life Support Organization (ELSO) registered 5123 COVID-19-associated ECMO therapies in 2020 and 7521 in 2021 worldwide [2]. In Germany, 4279 COVID-19-positive patients required ECMO therapy between January 2020 and September 2021.This national analysis of VV ECMO therapy revealed a mortality rate of 65.9% and a high incidence of complications [3].

A meta-analysis by Zangrillo et al. analyzed the complications of ECMO therapy, showing associated complications such as acute renal failure, bacterial pneumonia, hemorrhage, oxygenator dysfunction, sepsis, liver dysfunction, limb ischemia, stroke, and coagulation disorders [4]. In contrast to these well-examined associated complications, evidence on the combination of ECMO therapy and polyuria [5,6,7] or COVID-19 and polyuria is limited [8,9]. During the pandemic, our study group observed clinical polyuria in VV ECMO patients, as well as Statlender et al. [10]. Whether polyuria is merely a symptom or a complication aggravating the patient’s condition remains to be determined.

The most common reason for polyuria in an intensive care setting are the use of diuretics and fluid administration. Diabetes insipidus provoked by the dysregulation of the antidiuretic hormone (ADH) is a less common cause. The ADH, also called vasopressin, is one of the most important regulators of body fluid homeostasis, produced by the posterior pituitary gland.

This study aims to investigate the incidence of polyuria in a cohort of severe acute respiratory syndrome coronavirus 2 (SARS-CoV-2)-positive patients undergoing VV ECMO therapy and to analyze the potential causes and complications associated with polyuria.

## 2. Materials and Methods

### 2.1. Study Design

The study received ethical approval from the local ethics committee (Ethics committee of the Medical Department of the Goethe University Frankfurt, Germany; vote number: 20-643). All methods were performed in accordance with the relevant guidelines and regulations.

The inclusion criteria of this single-center retrospective study were as follows: patients aged ≥ 18, polymerase chain reaction (PCR)-confirmed diagnosis of SARS-CoV-2, and diagnosed ARDS and ECMO therapy. Patients were excluded if they required renal replacement therapy, veno-arterial ECMO (VA ECMO) therapy, or if data could not be collected up to 7 days after initiation, e.g., due to transfer to another hospital. The sample size was not calculated due to the study design. All patients who met the inclusion criteria were included.

Patients were divided into polyuric (PU) and non-polyuric (NPU) groups based on urine production after VV ECMO initiation. Polyuria was defined as an average urine production ≥3000 mL/d during the seven days following initiation [11]. Various parameters were recorded, including diuresis, fluid intake, excretion, fluid balance, VV ECMO flow within 24 h, cannula size and insertion site, serum levels of urea, creatinine, sodium, lactate, glucose, and procalcitonin. The in-house mortality rate was gathered for the respective length of the hospital stay. These parameters were assessed two days prior to VV ECMO initiation, on the day of initiation, and daily for seven days after initiation. For the documentation of the intensive care unit (ICU) stay, MetaVision ICU (Metavision 5.4, iMDsoft^®^, Tel Aviv, Israel) was used as the patient data management system. The fluid intake consisted of all parenteral and enteral administered fluids including continuous intravenous fluids and blood products, e.g., red blood cell concentrates. Excretion included reflux, feces, blood loss, and other secretions, e.g., chest drains. Plasma osmolality was calculated from the sodium, urea, and glucose results following a common formula with similar results to direct osmometry (with a known deviation of 1–2%) [12].

### 2.2. Statistical Analysis

Statistical analysis was performed with BiAS (BiAS version 11.12, Frankfurt, Germany). *p*-values < 0.05 were considered significant. If not described otherwise, data were expressed as arithmetic mean ± standard deviation. Graphics were designed using GraphPad Prism version 10.0.1 (GraphPad Software, Boston, MA, USA).

Spearman correlation was used to analyze the correlation between diuresis and ECMO flow, creatinine, and plasma osmolality from the day of ECMO initiation until day 7. A correlation was considered relevant, if the correlation coefficient was r ≥ 0.4. The VV ECMO flow rates, fluid intake, fluid balance, plasma osmolality, blood levels of creatinine, procalcitonin, lactate, urea, and sodium of the PU and NPU group were compared using Mann–Whitney U test due to non-Gaussian distribution.

The sub-analyses of diuresis, blood levels of urea, sodium, and plasma osmolality prior (day −2) to and after VV ECMO initiation (means of day 1 until day 7) were compared using the dependent sample *t*-test.

Fisher’s exact test was used to compare the groups regarding the use of vasopressin, norepinephrine, and diuretics.

### 2.3. Technical Characteristics

VV ECMO treatment was performed either with a rotaflow I^®^, rotaflow II^®^ (Getinge AB, Gothenburg, Sweden), or cardiohelp^®^ (Getinge AB, Gothenburg, Sweden) ECMO system. The cannulas used were manufactured by Maquet Cardiopulmonary GmbH (Rastatt, Germany) or Medtronic (Dublin, Ireland).

## 3. Results

**Figure 1:**
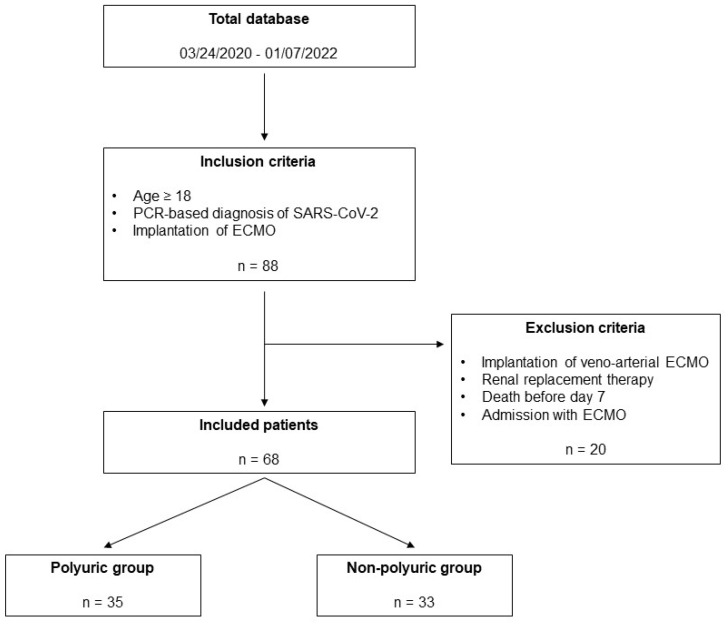
Study flow chart.

During the study period between 24 March 2020 and 7 January 2022, a total of n = 68 patients were treated at the COVID intensive care unit (Figure 1).

### 3.1. Patients’ Characteristics

In this study, 79% of the included patients were male and 21% were female. The mean age on the day of VV ECMO initiation was 52 ± 12 years and the mean body weight was 95 ± 18 kg. Table 1 illustrates the patients’ characteristics.

### 3.2. Statistical Results

**Figure 2:**
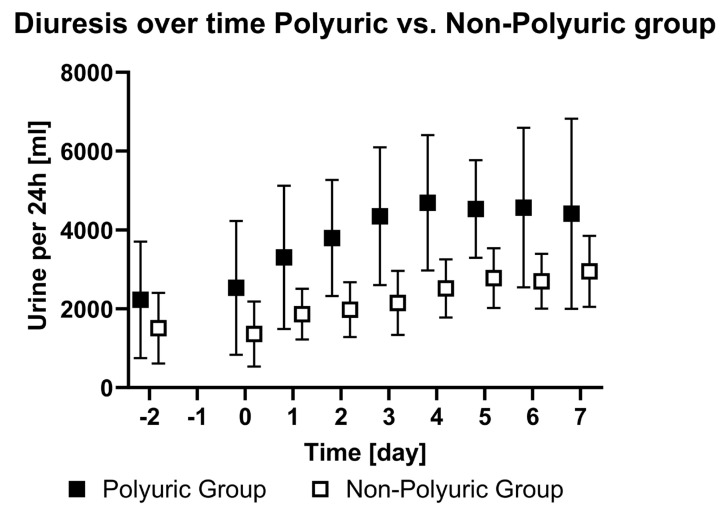
Diuresis over time in the polyuric vs. non-polyuric group. Mean amounts of urine per 24 h from two days prior to ECMO implantation (day -2), the day of ECMO implantation (day 0), and until seven days after ECMO implantation (day 7). No data were acquired on the day before ECMO implantation (day -1).

Following ECMO initiation, 51.5% (n = 35) of patients displayed polyuria between day one and day seven following ECMO initiation. Table A1 and Table A2 within Appendix A present the changes in the recorded parameters during the study period for the PU and NPU groups. Figure 2 illustrates the diuresis over time from day -2 to day 7 of the two groups. No relevant correlations were observed between diuresis and plasma osmolality (r = −0.19), diuresis and VV ECMO flow (r = 0.13), or diuresis and creatinine (r = −0.17).

**Figure 3:**
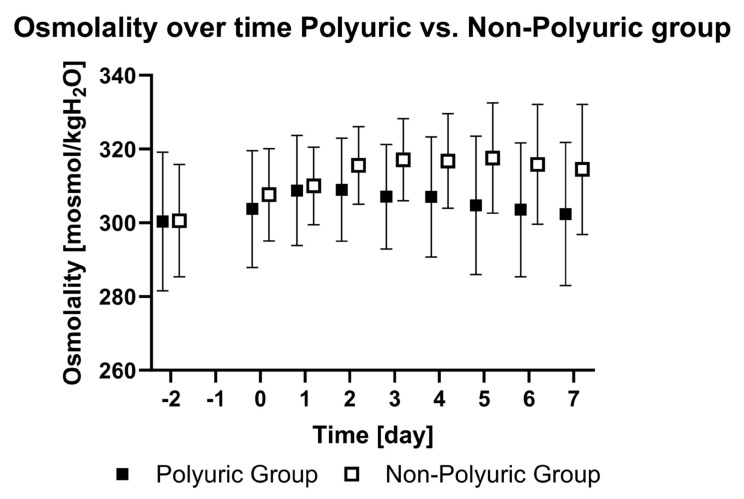
Osmolality over time in the polyuric vs. non-polyuric group. Mean plasma osmolality (calculated) from two days prior to ECMO implantation (day -2), the day of ECMO implantation (day 0), and until seven days after ECMO implantation (day 7). No data were acquired on the day before ECMO implantation (day -1).

Plasma osmolality and sodium levels increased after VV ECMO initiation compared to day -2 (*p* < 0.01). The NPU group showed a higher increase in plasma osmolality (*p* < 0.01) compared to the PU group (Figure 3).

**Figure 4:**
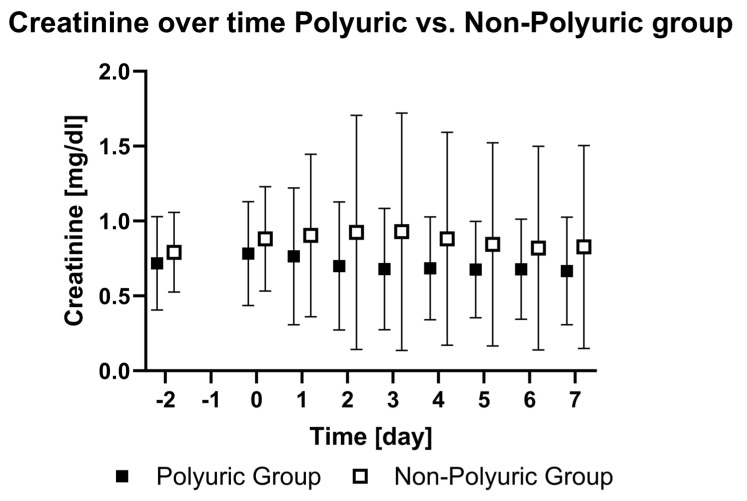
Creatinine over time in the polyuric vs. non-polyuric group. Mean plasma creatinine levels from two days prior to ECMO implantation (day -2), the day of ECMO implantation (day 0) until the seventh day after ECMO implantation (day 7). No data were acquired on the day before ECMO implantation (day -1).

The levels of creatinine did not change during the observation period. In the NPU group, creatinine (*p* < 0.01) levels were higher compared to the PU group (Figure 4). The urea levels did not increase in the sub-analyses from day -2 to day 7 (*p* = 0.62), but a difference was observed between the two groups, with increasing levels in the NPU group (*p* < 0.01).

The fluid intake was higher (PU: 4842 mL/24 h vs. NPU: 3226 mL/24 h) within the PU group (*p* < 0.01). The fluid balance differed between the groups (*p* = 0.01). The balance of the PU group was negative while the balance of the NPU group was positive (−95 mL/24 h vs. +174 mL/24 h).

There was no difference regarding the use of vasopressin (*p* = 0.29) and norepinephrine (*p* = 0.5) between the groups. Of all patients, 16% received vasopressin at some time during the study period (n = 7 within PU and n = 4 within NPU). No patient received vasopressin on more than three consecutive days. Every patient within the PU and 94% of the NPU received norepinephrine. Furthermore, 68.6% of the PU and 93.9% of NPU received diuretics (loop diuretics or acetazolamide) at some point during the observation period. There was no difference between the groups (*p* = 0.25).

There was no difference in the hospital mortality rate between the PU group (60%) and the NPU group (60.6%).

### 3.3. Technical Results

All patients were catheterized at two different vessels. The cannula diameter varied from 17 to 25 French according to vessel size and cannulation site. The most common cannula combination was 23 (drainage cannula) and 21 (return cannula) french (35.3%). Table 2 illustrates the different cannulation sites.

**Figure 5:**
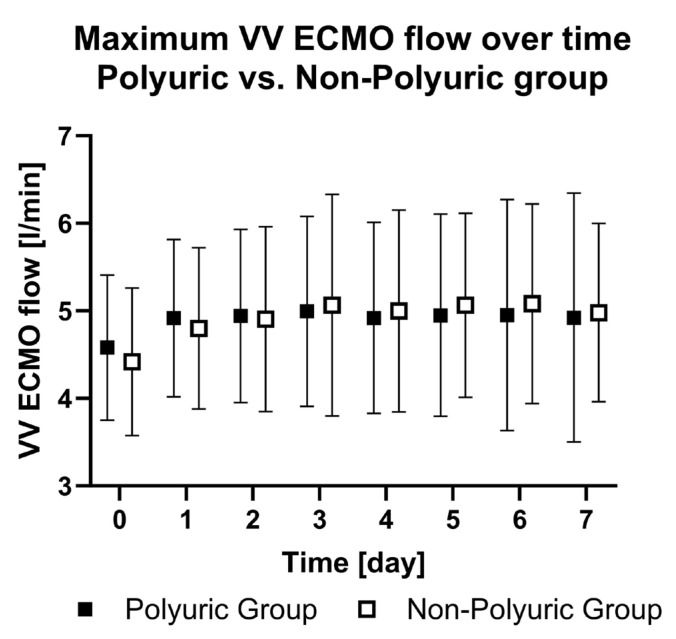
Maximum VV ECMO flow over time in the polyuric vs. non-polyuric group. Daily VV ECMO flow from day of ECMO implantation (day 0) until the seventh day after ECMO implantation (day 7).

The maximum VV ECMO flow rate remained constant at a median of 5 L/min throughout the observation period (Figure 5) and did not differ between the PU and NPU group (*p* = 0.82).

## 4. Discussion

The present study conducted at an ELSO member ECMO center demonstrated the high rate of polyuria among patients with COVID-19-associated ARDS who underwent ECMO therapy. The study indicates that polyuria did not have an impact on mortality and, although the electrolyte and volume status parameters differed between the PU and NPU patients, no relevance in mortality or the lengths of stay were observed.

While several complications and side effects of ECMO therapy have been published, there are only a few case reports available regarding polyuria [5,6,7]. At the beginning of our study, no reports specifically focused on COVID-19 patients receiving ECMO therapy were available. However, Statlender et al. recently published their findings about polyuria associated with the treatment of COVID-19 VV ECMO patients with vasopressin [10]. In their retrospective, single-center study, 12 of n = 37 COVID-19 patients undergoing VV ECMO treatment experienced episodes of diabetes insipidus, defined as a combination of polyuria (≥2.5 L/24 h), plasma osmolality ≥ 275 mOsm/kg, and urine osmolality ≤ 300 mOsm/kg. When urine osmolality data were unavailable, diabetes insipidus was classified as probable or possible based on sodium levels. Following the discontinuation of vasopressin, 91.6% of these patients experienced episodes of diabetes insipidus. Notably, according to our ICU’s standard operating procedure, vasopressin is administered only in cases of severe shock, in addition to norepinephrine at doses of ≥0.2 ug/kg/min. However, our current dataset did not reveal significant differences in the use of vasopressin between the groups, making it difficult to ascertain a direct link between vasopressin discontinuation and polyuria. Furthermore, within the range of mean arterial pressure from 80 to 170 mmHg, kidney autoregulation ensures constant filtration rates, suggesting that the increase in urine production cannot solely be attributed to the mean arterial pressure. In all patients, the mean arterial pressure was adjusted using norepinephrine as required.

The arithmetic mean of the daily urine output was determined as the main parameter in the current trial. Applying the criteria set forth by Statlender et al. [10] to our dataset, forty-nine out of the sixty-eight included patients would have met the criteria for probable diabetes insipidus at some point during the observation period. Given that this represents 72.1% of the entire cohort, the criteria for defining probable diabetes insipidus may appear liberal.

Although the fluid intake was notably higher in the PU group, we do not attribute this solely to the cause of polyuria. The negative balance observed in the PU group indicates a substantially elevated urine production compared to the NPU group, suggesting that the increased fluid intake may be a consequence rather than a cause of polyuria. It is plausible that the higher fluid intake in the PU group is necessary to maintain adequate intravascular volume and ensure regular ECMO flow.

Additionally, it is worth noting that, aside from defining polyuria based on specific daily urine output volumes, there are also definitions based on urine osmolality. However, neither our study nor the study by Statlender et al. measured urine osmolality daily [10]. Hence, future investigations into the impact of VV ECMO therapy on body fluid homeostasis should consider incorporating regular measurements of urine osmolality to provide a comprehensive understanding of polyuria dynamics.

COVID-19 presents with various symptoms, and there is evidence suggesting an influence on different hormonal axes [13]. Sheikh et al. reported a case of diabetes insipidus in a COVID-19 patient, which might have been caused by hypophysitis [8]. Thus, the SARS-CoV-2 infection itself may directly affect antidiuretic hormone (ADH) secretion. However, considering our severely affected patient population, renal failure is a more likely manifestation of COVID-19 [14,15].

ADH secretion is usually stimulated by an increase in plasma osmolality [12]. There are several regulating factors, including the volume and pressure differences mediated by baroreceptors and volume receptors in the vessels and the right atrium [16]. Based on these considerations, we hypothesize that VV ECMO treatment may influence these baroreceptors and volume receptors, particularly those in the right atrium, and alter the body’s fluid homeostasis through this pathway. This theory is also supported by a case report of polyuria under VA ECMO therapy in a non-COVID-19 patient [5]. Further investigation requires the measurement of plasma ADH levels.

Our study demonstrates an increase in diuresis and plasma osmolality after VV ECMO initiation. One possible explanation for this finding is that VV ECMO treatment caused polyuria by activating baroreceptors and concomitant “dehydration”, which is reflected in the increased plasma osmolality. Plasma osmolality is a highly regulated parameter of the body’s fluid homeostasis [11]. Therefore, a rise in osmolality in association with an increasing urine output is significant. Normally, an increase in plasma osmolality triggers ADH secretion and leads to an “anti-diuretic” state with a decreased urine output. Although we observed a relevant increase in both parameters in patients undergoing VV ECMO treatment, there was no significant correlation between plasma osmolality and diuresis. The PU group exhibited lower plasma osmolality than the NPU group. We consider this as a possible cause for polyuria since lower plasma osmolality leads to lower ADH secretion. Whether this is a coincidence, or whether there is a causal link, requires further discussion and research. In line with the higher plasma osmolality, sodium levels within the NPU group were also higher. This can be explained by the important role of sodium in determining plasma osmolality.

The creatinine and urea levels were higher in the NPU group and did not show significant alterations during the study period. Levels in both groups were within the physiological ranges. Therefore, it is unlikely that the difference is due to actual “better” renal function, but rather to the limitations of creatinine and urea as parameters for renal function in patients with polyuria. More important is the finding that polyuria did not impair renal function, which, alongside age, is one of the most prevalent risk factors for mortality during ECMO therapy [3].

## 5. Future Directions and Limitations

This study is constrained by its retrospective design and the single-center setting. Data availability for diuresis before ECMO initiation was limited due to patient transfers from various wards and hospitals. Urine samples were neither taken daily nor in a structured manner during the study period. The investigation of urine samples might provide further information to identify the cause of polyuria. Although diuretics were carefully administered to achieve a negative fluid balance and maintain acid–base equilibrium, it is noteworthy that there was no significant difference observed between the groups. The multifactorial nature of polyuria in the context of complex intensive care treatment suggests potential confounding factors. While exploring plasma ADH levels for further insights, obtaining a reliable dataset for this parameter would pose logistical challenges, necessitating significant efforts such as cooling probe tubes for accurate measurement. Copeptin has been described as a surrogate marker of ADH with high sensitivity and specificity [17]. Contrary to ADH, Copeptin is stable in serum probes under room temperature.

A correlation of ventilation parameters and haemodynamic changes before and after ECMO initiation could lead to further insights of the causes of polyuria since right ventricular function might improve under lung protective ventilation. Renal function could also increase under right ventricular relief.

Shekar et. al. identified an increased volume of distribution as one of the most common mechanisms for pharmacokinetic alternations in ECMO therapy [18]. The impact of polyuria on homeostasis might also lead to additional pharmacokinetic changes. Further research in this field could lead to therapeutic consequences, e.g., dosing of antibiotics.

## 6. Conclusions

Diuresis and plasma osmolality increased during VV ECMO treatment. Notably, polyuria did not impact mortality nor exacerbate renal impairment or failure. The etiology of these effects remains uncertain, whether attributable to VV ECMO therapy, COVID-19, or other ICU-related factors. Future investigations into VV ECMO therapy’s fluid homeostasis should prioritize the frequent monitoring of urine osmolality and plasma ADH levels for comprehensive understanding.

## Figures and Tables

**Table 1:** Patients’ characteristics.

Characteristics	Females n (%)	Males n (%)	Age [years]	Weight [kg]
Total patients	14 (21)	54 (79)	52 ± 12	95 ± 18
PU	5 (14)	30 (86)	49 ± 13	94 ± 19
NPU	9 (27)	24 (73)	54 ± 10	95 ± 15

**Table 2:** Cannulation site.

Cannulation Site	Femoral Right and Left (V_f_ − V_f_)	Femoral Right and Internal Jugular Right (V_f_ − V_j_)	Internal Jugular Right and Femoral Left (V_f_ − V_j_)
All patients n (%)	50 (73.5)	16 (23.5)	2 (3)
PU	28 (80)	7 (20)	0
NPU	22 (66.7)	9 (27.3)	2 (6)

## Data Availability

The datasets used and analyzed during the current study are available from the corresponding author on reasonable request.

## List of Abbreviations

ARDS	acute respiratory distress syndrome
ADH	antidiuretic hormone
COVID-19	coronavirus disease 2019
ELSO	Extracorporeal Life Support Organization
ICU	intensive care unit
NPU	non-polyuric
PCR	polymerase chain reaction
PU	polyuric
SARS-CoV-2	severe acute respiratory syndrome coronavirus 2
VA ECMO	veno-atrial extracorporeal membrane oxygenation
VV ECMO	veno-venous extracorporeal membrane oxygenation

## Appendix A

**Table A1:** Parameters of the polyuric and non-polyuric group part 1. Overview of the first part of the results in the polyuric (PU) and non-polyuric (NPU) group. Diuresis within 24 h, average ECMO flow rate, creatinine, and plasma osmolality levels are shown from two days prior to ECMO implantation (day -2), on the day of ECMO implantation (day 0), and until the seventh day after ECMO implantation (day 7). It also illustrates the statistical results of the Mann–Whitney U test (*) and the dependent sample *t*-test (#).

Group	Parameters	Day −2	Day 0	Day 1	Day 2	Day 3	Day 4	Day 5	Day 6	Day 7	*p*-Value #	*p*-Value *
PU	Diuresis [mL/24 h]	2225 ± 1443	2605 ± 1640	3303 ± 1789	3793 ± 1451	4347 ± 1722	4689 ± 1693	4529 ± 1220	4566 ± 1993	4411 ± 2377	<0.01	-
NPU	Diuresis [mL/24 h]	1504 ± 872	1358 ± 814	1864 ± 631	1977 ± 684	2148 ± 799	2514 ± 725	2777 ± 748	2698 ± 683	2947 ± 886		
PU	ECMO flow rate [L/min]	-	4.6 ± 0.8	4.9 ± 0.9	4.9 ± 1.0	5.0 ± 1.1	4.9 ± 1.1	4.9 ± 1.1	5.0 ± 1.3	4.9 ± 1.4	-	0.82
NPU	ECMO flow rate [L/min]	-	4.4 ± 0.8	4.8 ± 0.9	4.9 ± 1.0	5.1 ± 1.2	5.0 ± 1.1	5.1 ± 1.0	5.1 ± 1.1	5.0 ± 1.0		
PU	Creatinine [mg/dL]	0.72 ± 0.3	0.78 ± 0.34	0.76 ± 0.45	0.7 ± 0.42	0.68 ± 0.4	0.68 ± 0.34	0.7 ± 0.32	0.68 ± 0.33	0.67 ± 0.35	-	<0.01
NPU	Creatinine [mg/dL]	0.79 ± 0.26	0.88 ± 0.34	0.9 ± 0.53	0.92 ± 0.77	0.93 ± 0.78	0.88 ± 0.7	0.84 ± 0.67	0.82 ± 0.67	0.83 ± 0.67		
PU	Plasma osmolality [mosmol/kgH_2_O]	300 ± 18	304 ± 16	309 ± 15	309 ± 14	307 ± 14	307 ± 16	305 ± 19	303 ± 18	302 ± 19	<0.01	<0.01
NPU	Plasma osmolality [mosmol/kgH_2_O]	300 ± 15	308 ± 12	310 ± 10	316 ± 10	317 ± 11	317 ± 13	318 ± 15	316 ± 16	315 ± 17		

**Table A2:** Parameters of the polyuric and non-polyuric group part 2. Overview of the second part of the results in the polyuric (PU) and non-polyuric (NPU) group. Levels of urea, sodium, lactate, and procalcitonin, as well as fluid intake and fluid balance are shown from two days prior to ECMO implantation (day -2), on the day of ECMO implantation (day 0), and until the seventh day after ECMO implantation (day 7). It also illustrates the statistical results of the Wilcoxon–Mann–Whitney U-test (*) and the dependent sample *t*-test (#).

Group	Parameters	Day −2	Day 0	Day 1	Day 2	Day 3	Day 4	Day 5	Day 6	Day 7	*p*-Value #	*p*-Value *
PU	Urea [mg/dL]	44 ± 28	45 ± 21	50 ± 30	48 ± 33	46 ± 34	44 ± 34	44 ± 32	42 ± 30	42 ± 29	0.62	<0.01
NPU	Urea [mg/dL]	48 ± 27	53 ± 25	57 ± 24	58 ± 26	60 ± 30	61 ± 32	60 ± 34	60 ± 36	61 ± 38		
PU	Sodium [mmol/L]	143 ± 8	145 ± 7	146 ± 6	147 ± 5	146 ± 5	146 ± 6	145 ± 7	144 ± 7	144 ± 8	<0.01	<0.01
NPU	Sodium [mmol/L]	142 ± 6	148 ± 5	146 ± 5	148 ± 4	149 ± 4	149 ± 5	149 ± 5	149 ± 6	148 ± 6		
PU	Lactate [mg/dL]	13 ± 3	14 ± 8	13 ± 5	11 ± 4	10 ± 4	9 ± 3	8 ± 2	9 ± 3	8 ± 3	-	<0.01
NPU	Lactate [mg/dL]	15 ± 4	17 ± 7	14 ± 5	12 ± 3	12 ± 4	10 ± 4	10 ± 3	9 ± 3	9 ± 3		
PU	Procalcitonin [ng/mL]	0.56 ± 0.7	0.86 ± 1.23	1.0 ± 1.3	0.79 ± 1.02	0.86 ± 1.3	0.79 ± 1.1	0.6 ± 0.75	0.47 ± 0.49	0.45 ± 0.42	-	0.41
NPU	Procalcitonin [ng/mL]	0.58 ± 0.79	0.92 ± 0.92	1.46 ± 2.06	1.08 ± 1.52	2.28 ± 8.66	0.59 ± 0.61	0.49 ± 0.51	0.5 ± 0.77	0.44 ± 0.59		
PU	Fluid intake [mL/24 h]	3296 ± 1551	3816 ± 2445	4402 ± 1846	4429 ± 1495	4684 ± 2086	5002 ± 1718	4938 ± 1748	5064 ± 2208	5375 ± 2346	-	<0.01
NPU	Fluid intake [mL/24 h]	2385 ± 1275	2964 ± 1605	3407 ± 1049	2985 ± 929	3355 ± 922	3065 ± 786	3036 ± 800	3291 ± 1034	3443 ± 1183	-	-
PU	Fluid balance [mL/24 h]	604 ± 920	821 ± 1333	331 ± 1447	23 ± 1126	−565 ± 1528	−276 ± 1178	−201 ± 1334	−128 ± 1438	153 ± 1343	-	0.01
NPU	Fluid balance [mL/24 h]	307 ± 1032	1161 ± 1175	863 ± 1117	261 ± 1088	327 ± 979	90 ± 1175	−195 ± 1019	75 ± 1112	−202 ± 1368	-	-
